# Can cannabis kill? Characteristics of deaths following cannabis use in England (1998–2020)

**DOI:** 10.1177/02698811221115760

**Published:** 2022-08-10

**Authors:** Kirsten L Rock, Amir Englund, Stephen Morley, Kathleen Rice, Caroline S Copeland

**Affiliations:** 1 Centre for Pharmaceutical Medicine Research, Institute of Pharmaceutical Sciences, King’s College London, London, UK; 2 Department of Addictions, Institute of Psychiatry, Psychology & Neuroscience, King’s College London, South London and Maudsley NHS Foundation Trust, London, UK; 3 Toxicology Unit, Leicester Royal Infirmary, Leicester, UK; 4 National Programme on Substance Abuse Deaths, London, UK

**Keywords:** Cannabis, cannabinoids, Δ9-tetrahydrocannabinol, THC, toxicity, drug-related death

## Abstract

**Background:**

Cannabis is the most widely used illegal drug but is rarely considered a causal factor in death.

**Aims:**

This study aimed to understand trends in deaths in England where cannabinoids were detected at post-mortem, and to evaluate the clinical utility of post-mortem cannabinoid concentrations in coronial investigations.

**Methods:**

Deaths with cannabinoid detections reported to the National Programme on Substance Abuse Deaths (NPSAD) were extracted and analysed.

**Results:**

From 1998 to 2011, on average 7% of all cases reported to NPSAD had a cannabinoid detected (*n* = 110 deaths per year), rising to 18% in 2020 (*n* = 350). Death following cannabis use alone was rare (4% of cases, *n* = 136/3455). Traumatic injury was the prevalent underlying cause in these cases (62%, *n* = 84/136), with cannabis toxicity cited in a single case. Polydrug use was evident in most cases (96%, *n* = 3319/3455), with acute drug toxicity the prevalent underlying cause (74%, *n* = 2458/3319). Cardiac complications were the most cited physiological underlying cause of death (4%, *n* = 144/3455). The median average Δ9-tetrahydrocannabinol post-mortem blood concentrations were several magnitudes lower than previously reported median blood concentrations in living users (cannabis alone: 4.3 µg/L; cannabis in combination with other drugs: 3.5 µg/L).

**Conclusions:**

Risk of death due to cannabis toxicity is negligible. However, cannabis can prove fatal in circumstances with risk of traumatic physical injury, or in individuals with cardiac pathophysiologies. These indirect harms need careful consideration and further study to better elucidate the role cannabis plays in drug-related mortality. Furthermore, the relevance of cannabinoid quantifications in determining cause of death in coronial investigations is limited.

## Introduction

The cannabis plant (*Cannabis sativa* L.) contains more than 60 ligands which bind to the cannabinoid receptors type 1 (CB1) and type 2 (CB2) (Dale and Haylett, 2009). The main intoxicating component of cannabis, Δ9-tetrahydrocannabinol (THC), is a partial agonist of the CB1 receptor and mediates most of the central nervous system (CNS) effects observed following cannabis use (Kendall and Yudowski, 2016). Cannabis intoxication is dose dependent and can affect memory, attention and psychomotor performance at low doses, whereas higher doses can trigger paranoid ideations, visual and auditory hallucinations and potential cardiovascular effects (increased heart rate and blood pressure, arrhythmias) (Breijyeh et al., 2021).

According to the Office for National Statistics (ONS), cannabis is the most commonly used illegal drug in England and Wales: in 2019/20, 29.6% of people aged 16–59 had used cannabis at least once during their lifetime (ONS, 2020). Despite this use prevalence, cannabis is rarely considered a significant contributory or causal factor in drug-related deaths unless in a trauma setting, for example, road traffic collision (RTC) (Holland et al., 2011). Indeed, drug poisoning deaths in England and Wales involving cannabis have remained low since recordings began in 1993, averaging 0.6 deaths per million people (ONS, 2020). However, cannabis does negatively affect driving performance by impairing cognitive and motor function with drivers exhibiting delayed reaction time, greater lane position variability and reduced attention (Hartman and Huestis, 2013). The Driving Under the Influence of Drugs, Alcohol and Medicines (DRUID) project in Europe identified cannabis as the second most common psychoactive substance detected in RTC (succeeded only by alcohol) and its prevalence ranged between 0.5%–2.2% in serious non-fatal accidents and 0.0%–1.8% in fatal accidents (EMCDDA, 2012).

In this study, deaths following cannabis use reported to the National Programme on Substance Abuse Deaths (NPSAD) have been analysed to understand recent trends in deaths and decedent demographics, and to evaluate the clinical utility of post-mortem cannabis concentrations in coronial investigations.

## Methods

### National Programme on Substance Abuse Deaths

NPSAD regularly receives reports from 88.0% of English coroners on deaths related to psychoactive drugs, as previously described (Oyekan et al., 2021). A death is referred to a coroner if it has an unknown cause, is violent or unnatural, sudden and unexplained, occurred during an operation or before the person came out of an anaesthetic, or potentially caused by an industrial disease or poisoning (www.gov.uk, 2020). Toxicology tests are requested depending upon individual case circumstances at the discretion of the coroner and consulting pathologist.

The King’s College London Biomedical & Health Sciences, Dentistry, Medicine and Natural & Mathematical Sciences Research Ethics Subcommittee confirmed (November 2020) that NPSAD does not require research ethics committee review as all subjects are deceased.

### Case identification

A retrospective study design identified all cases with THC and/or its metabolites (THC–COOH and THC–OH) detected that were reported from England by searching the entire NPSAD database (records received from 1997 to 22nd Apr 2021) in the post-mortem drug fields for the numerical code assigned to cannabis.

### Data analysis

*Software*: Data analysis and statistics (Spearman’s rank, Student’s *t*-test, Chi squared) were performed using IBM^®^ SPSS™ Statistics for Windows version 27 and Μicrosoft Excel 365.

2020 *projection*: The average time between death and coronial inquest conclusion where cannabis is present is 7–10 months. Further deaths occurring in 2020 are therefore anticipated to be reported to NPSAD. Based on jurisdiction reporting trends, the number of deaths with cannabinoid detections expected to be received by NPSAD has been projected.

*Cause of death*: Circumstances that lead to death are categorised on the death certificate issued by the coroner in the following manner:

Cause 1a: The immediate cause of death (and underlying if no 1b or 1c cited)
Cause 1b: Any disease/circumstances underlying Cause 1a
Cause 1c: Any disease/circumstance underlying Cause 1b
Cause 2: Any disease/circumstance that did not cause the death but contributed in some way

It is not a requirement for a Cause 1b, 1c or 2 to be cited for all deaths (www.gov.uk, 2020). Immediate and underlying cause of death were identified using these criteria.

*Deprivation scores*: The English Indices of Deprivation 2019 was used to obtain deprivation data (Ministry of Housing, Communities & Local Government, 2019).

## Results

**Figure 1.**
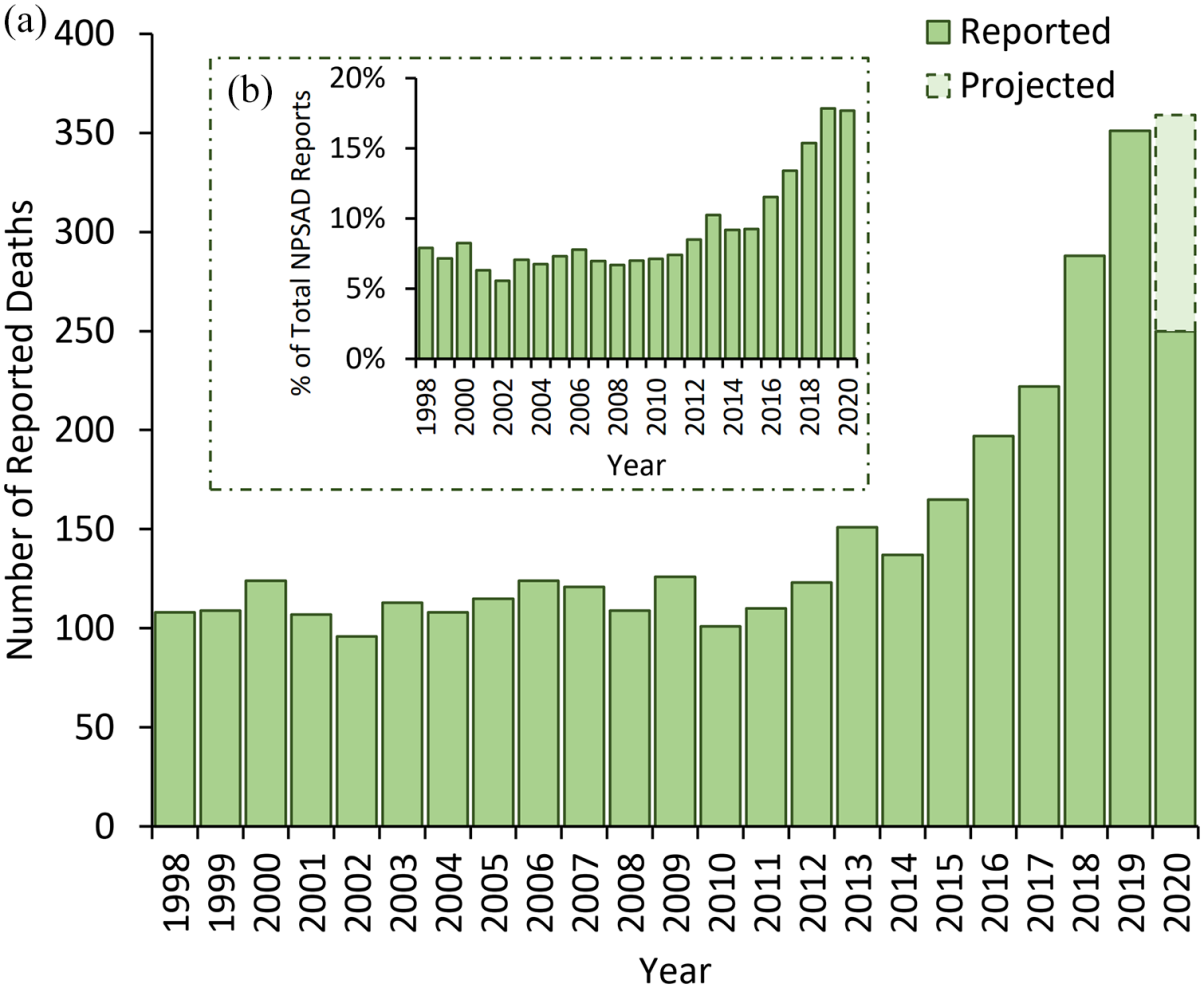
(a) Number of deaths reported to NPSAD from England (1998–2020) with cannabis detections at post-mortem. As the average period between death and conclusion of coronial inquests for drug-related deaths is 7–10 months, further deaths from 2020 are anticipated to be reported. The number of deaths projected to still be received (dashed bar area) has been calculated based upon these previous jurisdiction reporting trends. (b) Proportion of deaths with cannabis detections at post-mortem reported to NPSAD from England (1998–2020). When normalised against total NPSAD reporting in England over the same time period, the increase in deaths with cannabis detections remains, demonstrating that there has been a proportional rise in their occurrence.

**Figure 2.**
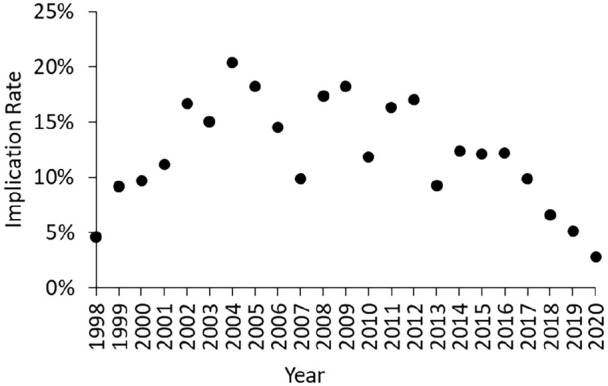
Implication rate of cannabis in deaths reported to NPSAD from England (1998–2020). Note that whilst the 2020 implication rate has been calculated, this is subject to change pending receiving additional reports.

Cannabinoids were detected in post-mortem tissue(s) of 3455 people who died in England and were reported to NPSAD by 22nd Apr 2021. An average of 110 deaths per year were reported from 1998 to 2011, but this has since risen with a total of over 350 deaths projected to be reported from 2020 (Figure 1(a)). This increase in prevalence is reflected when considering the percentage of all cases reported to NPSAD: in 1998–2011, an average of 7% of all NPSAD cases had evidence of recent cannabis use, which rose to 18% for those reported from 2020 (Figure 1(b)). Concurrently, the implication of cannabis as a cause of death sharply declined in the last 5 years of the study from an average 14% implication rate in 1998–2016 to 3% in 2020 (Spearman’s rank 2016–2020 *r* = −0.99; Figure 2).

### Cause and manner of death

Death following use of cannabis alone was rare (4% of cases, *n* = 136/3455). In these cases, traumatic injury was the most common underlying cause of death (62% of cases, *n* = 84/136); the majority of which were due to self-inflicted injuries (e.g. hanging, traumatic injury following intentional fall from a height) (55% of cases, *n* = 47/84) or RTCs (41% of cases, *n* = 35/84). In the remaining two cases the intent of the injury could not be determined, with the coroner returning an open verdict. Cannabis use itself was deemed the underlying cause of death in only 14 cases: in 13 of these cases cannabis use preceded immediate cause of death by cardiac failure (*n* = 9), aspiration (*n* = 1), cerebral haemorrhage (*n* = 1) or traumatic injury (*n* = 1). Cannabis toxicity was attributed as the sole underlying and immediate cause of death in one case. Here, the consulting pathologist noted a level of THC between 100 and 150 μg/L detected in the blood, with no medical illness or trauma evident upon post-mortem examination, although the decedent was reportedly a heavy cannabis user.

**Figure 3.**
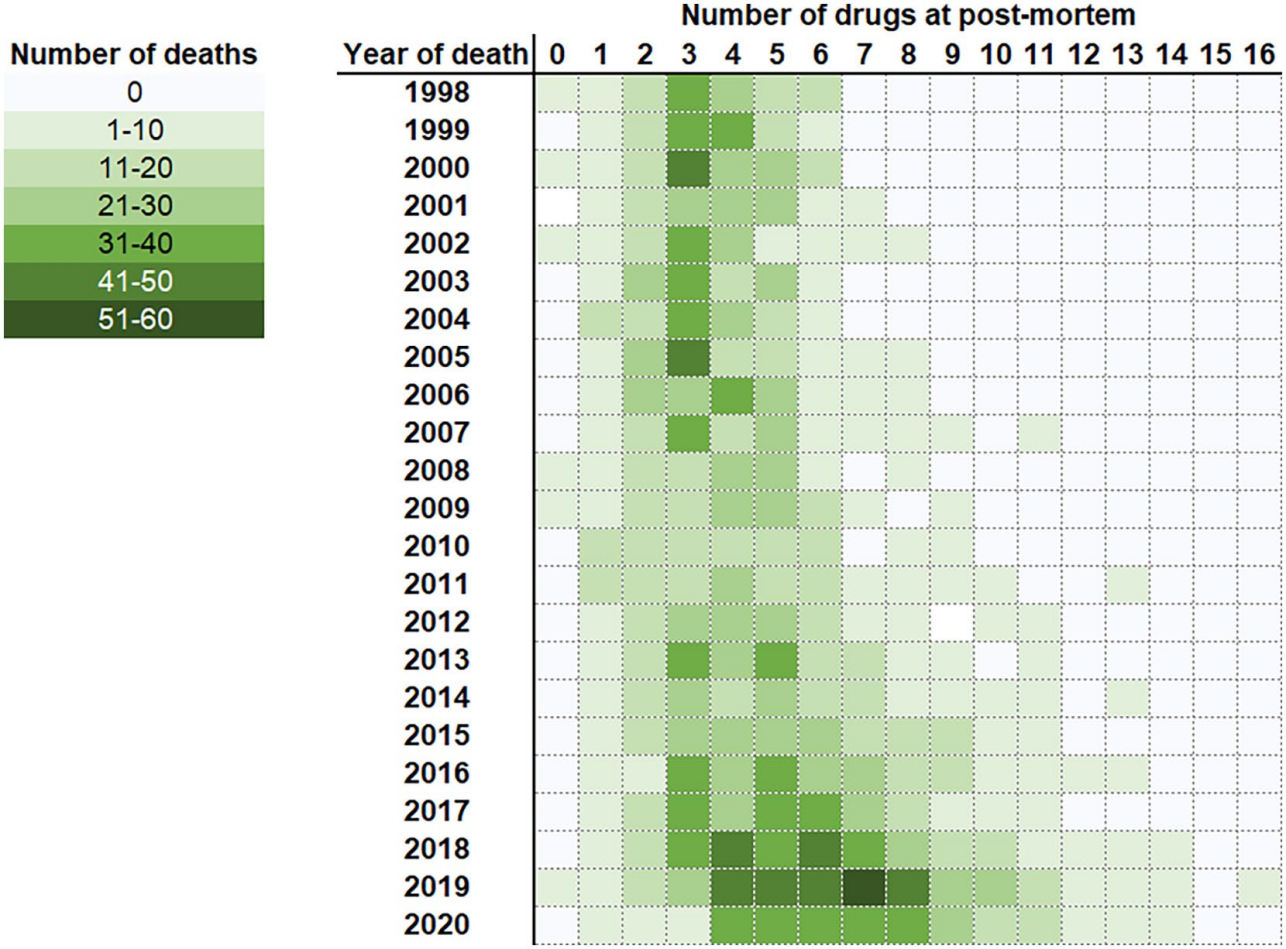
Number of drugs co-detected in cannabis cases by year. Note that whilst the 2020 data has been included, this is subject to change pending receiving additional reports.

Cannabis use in combination with other drugs was evident in most cases (96%; *n* = 3319/3455; Figure 3). Death due to acute drug toxicity was the most common underlying cause of death (74% of cases, *n* = 2458/3319). However, cannabis itself was rarely co-implicated in causing death with the other co-detected drugs (7% of cases, *n* = 228/3319). Traumatic injury featured as the underlying cause of death in 10% of these cases (*n* = 328/3319), with proportions of death due to self-inflicted injury (59%; *n* = 194/328) and RTCs (30%, *n* = 99/328) comparable to those where cannabis was detected alone.

Cardiac complications were the most cited physiological underlying cause of death (4% of cases, *n* = 144/3455). Cardiac disease (e.g. ischaemic heart disease, atherosclerosis/atheroma, myocarditis) was cited in 61% of these cases (*n* = 88/144), with morphological alterations of the cardiac structure (e.g. hypertrophy, fibrosis, myopathy) cited in 22% of these cases (*n* = 32/144).

Whilst the majority of cases were deemed accidental in nature, when delineating by polydrug use, significant proportions of decedents who had used cannabis alone were deemed to have died by suicide or where intent was undeterminable in comparison to total cases reported to NPSAD (Table 1, both *p* < 0.001).

**Table 1.** Manner of death of decedents who had used cannabis delineated by polydrug use.

Manner	Total cannabis cases		Cannabis only		Cannabis and other drugs		Total NPSAD
	Cases (*n*)	%	Cases (*n*)	%	Cases (*n*)	%	%
Natural	101	3	19	14	82	3	2
Accidental	2783	81	57	42	2726	82	72
Suicidal	277	8	36	27	241	7	15
Homicidal	13	<1	0	–	13	<1	<1
Undetermined	281	8	24	18	257	8	11

### Levels of cannabinoids

**Figure 4.**
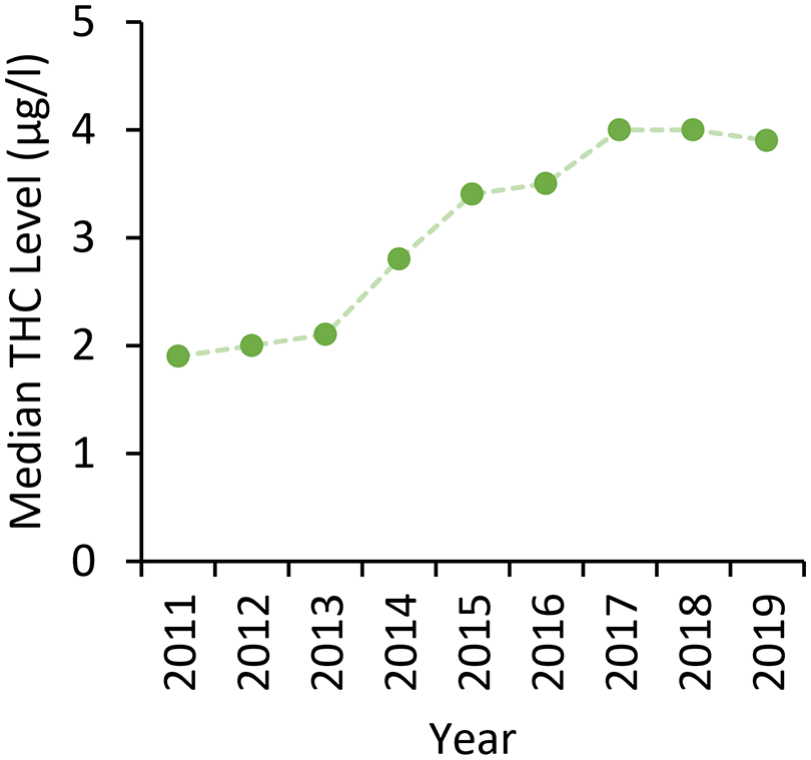
Median detected post-mortem levels of THC in cases where quantifications were performed; 2020 data has been excluded due to the low number of cases with THC quantifications provided at the time of writing.

In cases where cannabinoid levels in post-mortem blood were quantified (THC *n* = 782; THC–COOH *n* = 758 and THC–OH *n* = 117), the median concentrations detected were comparable whether cannabis had been used alone or in combination with other drugs, or when delineating by manner of death (Table 2). However, higher concentrations of all three cannabinoids were detected in accidental RTC cases in comparison to accidental overdoses due to drug toxicity. When considering levels of THC detected over time, in the later 10 years of the study, where quantification of cannabinoids was more routinely carried out (EMCDDA, 2021), there is evidence of small year-on-year increases (Figure 4).

**Table 2.** Median levels (μg/L) of cannabinoids detected in post-mortem blood samples by type of cannabis, manner of death and type of accidental death.

	Median blood level (μg/L)		
	THC	THC–COOH	THC–OH
Type of cannabis use			
Cannabis alone	4.3	16.4	1.8
Cannabis and other drugs	3.5	10.0	1.5
Manner of death			
Accidental	3.5	10.0	1.6
Drug toxicity	3.4	10.0	1.5
Road traffic collision	9.0	38.0	10.0
Suicidal	4.9	8.8	0.9
Undetermined	2.2	14	1.4

### Co-detected drugs

**Figure 5.**
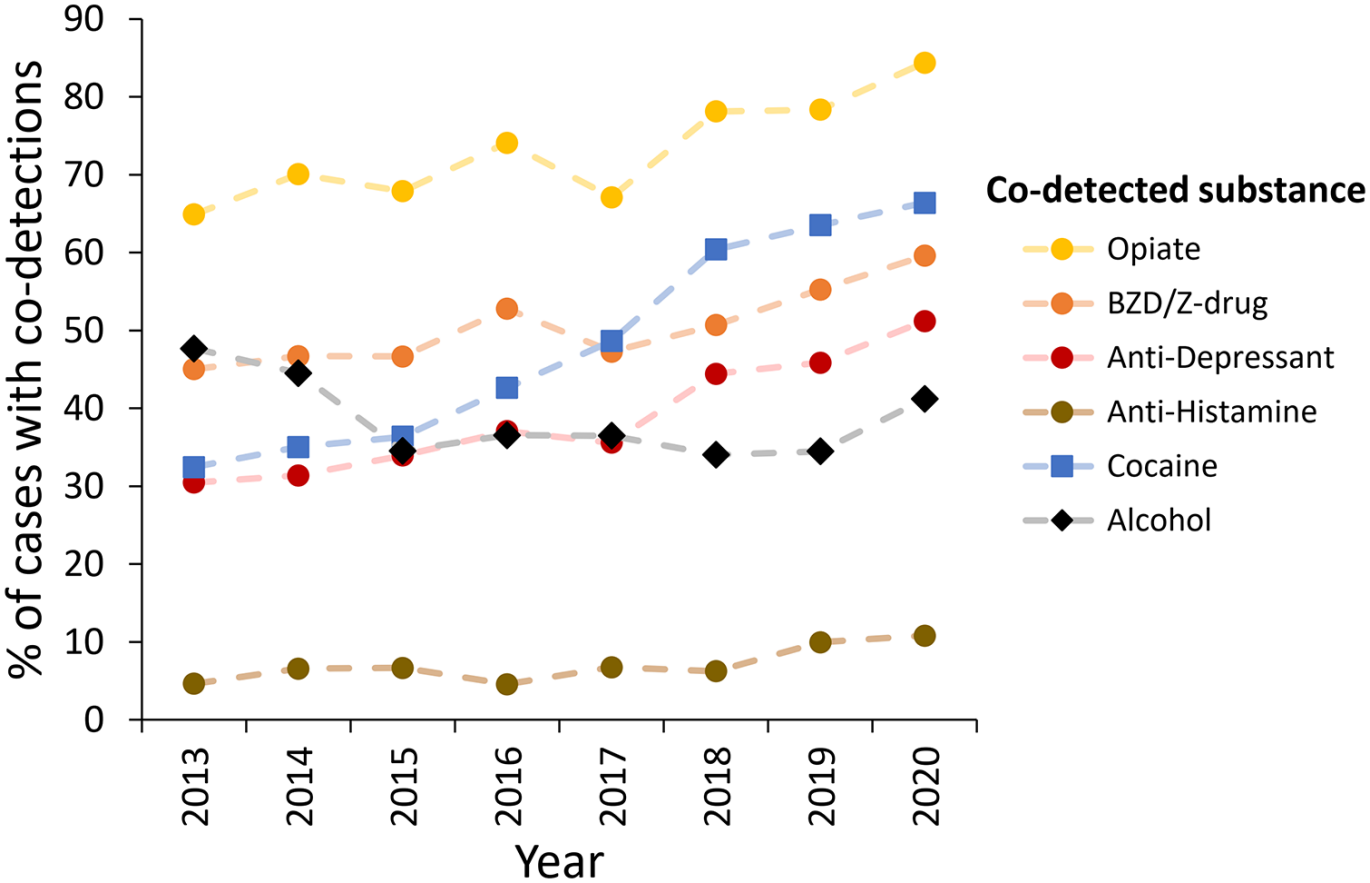
Proportion of cannabis cases with co-detected substances over time. Note that whilst the 2020 data has been included, this is subject to change pending receiving additional reports.

Throughout the course of the study the extent of polydrug use increased with an average of three or four drugs detected at post-mortem in deaths that occurred during 1998–2013, which increased to six or seven drugs in deaths that occurred during 2018–2020 (Figure 3). During the rising phase of overall number of cannabis deaths and polydrug use (2013–2020), concomitant increases in co-detections of CNS depressants (opiates, benzodiazepines/Z-drugs, anti-depressants, antihistamines); and cocaine was evident (Figure 5). Alcohol was co-detected in 39% of cases (*n* = 1358/3455; cases where alcohol was attributed to likely post-mortem production by the toxicologist (usually ⩽10 mg/dL) (O’Neal and Poklis, 1996) were excluded) and did not increase in prevalence over the course of the study (Spearman’s rank *r* = −0.07, Figure 5). Synthetic cannabinoids were co-detected in a small number of cases (*n* = 39).

### Demographics

**Figure 6.**
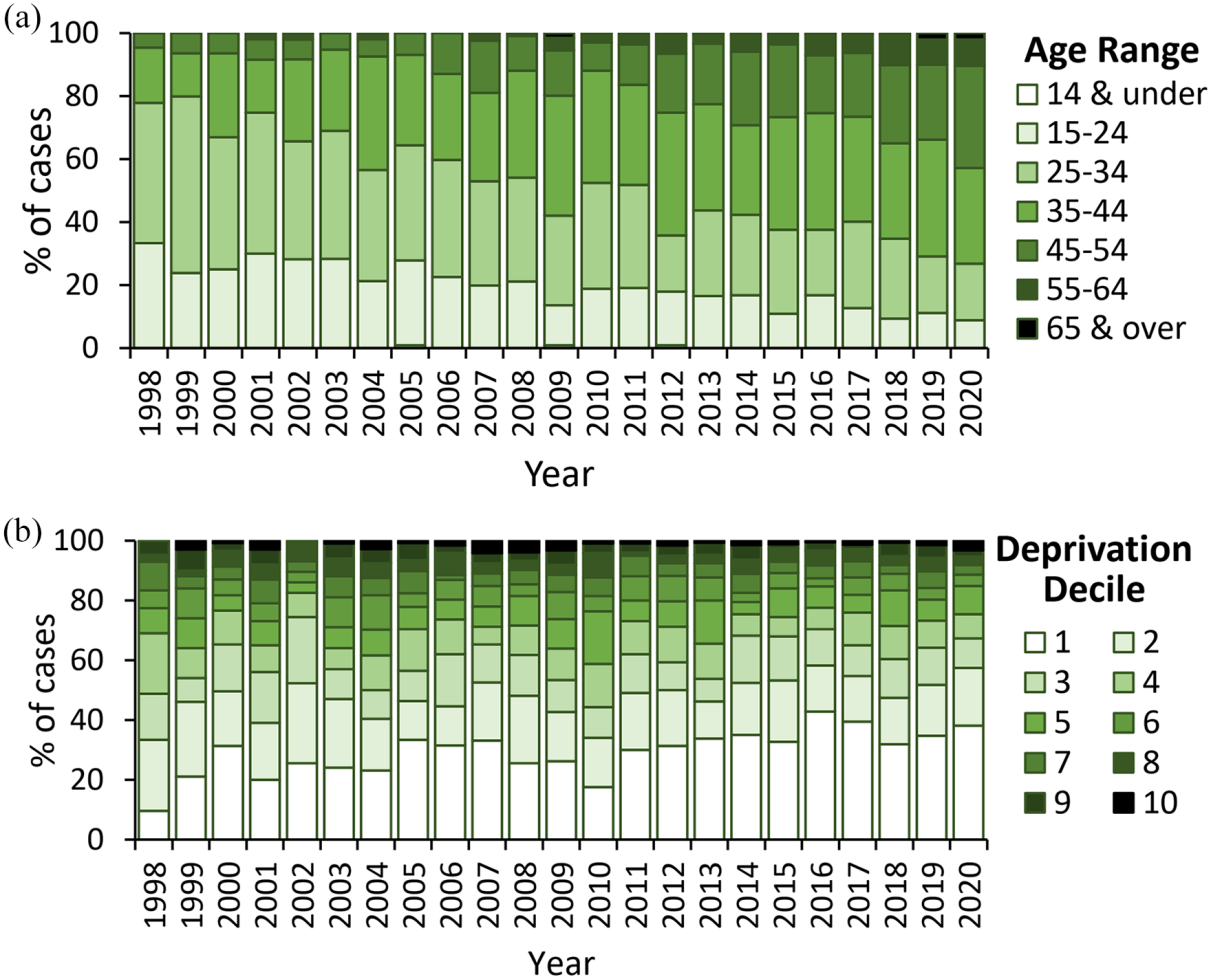
(a) Age at death and (b) decile of deprivation of decedents with cannabinoids detected at post-mortem. Note that whilst the 2020 data has been included, this is subject to change pending receiving additional reports.

The majority of decedents were male (85%) and had a known history of substance use disorder (69%). Age of decedents increased over time (Figure 6(a)), as did the proportion of decedents living in the most deprived areas of England (Figure 6(b); deprivation decile 1 – most deprived, 10 – least deprived).

## Discussion

Deaths due to cannabinoid detections have increased in England, with the number of reported deaths in 2020 more than twice of those reported 10 years earlier. As testing for cannabinoids at post-mortem has been a routine practice in the United Kingdom since the 1990s (EMCDDA, 2021), and the relative proportion of deaths with cannabinoid detections reported to NPSAD increased, it is unlikely an artefact due to increased testing or reporting. However, as overall use prevalence in the United Kingdom has not increased at the same rate (UK Government, 2021), this likely reflects increased use prevalence specifically in people who also use other substances with fatal consequences. Given the ongoing global debate regarding cannabis and its associated harms (Hussain et al., 2021), it is important to examine trends in these deaths to understand and interpret their impact.

### Risk of traumatic injury outweighs that of toxicity

Cannabis was the sole drug detected at post-mortem in only 4% of deaths. Traumatic injury was the prevalent underlying cause in these cases, with the citation of pathophysiological underlying causes comparatively rare and toxicity evident in only a single case.

Self-inflicted injuries comprised the greatest proportion of trauma-related deaths. There is clear evidence for a link between depression severity and suicidal ideation (Herrman et al., 2022), and there is growing evidence linking cannabis use and depression: chronic cannabis users have a higher incidence of depression diagnoses (Lev-Ran et al., 2014), with those who started using cannabis during adolescence at greatest risk (Gobbi et al., 2019). Over one-third of people with depression report using cannabis medicinally to manage their depressive symptoms (Kosiba et al., 2019), despite evidence that cannabis use is associated with poorer outcomes in recovery (Bahorik et al., 2017). However, a causal relationship between cannabis use and depression may be confounded by social and environmental risk factors for both substance use and mental disorders (Degenhardt et al., 2003) – risk factors likely significant for the decedents in this study as evidenced by their high rate of residence in socioeconomically deprived areas. Additionally, there are emerging links between cannabis use disorder and other mental disorders such as dissociation, a feature of psychosis (Ricci et al., 2021). Dissociation is associated with higher rates of self-harm and suicide attempts (Calati et al., 2017), which may explain in part the greater proportion of self-inflicted injuries.

Fatal injury following RTC accounted for almost all the remainder of trauma-related deaths. Cognitive impairments (e.g. reduced vigilance and control, extended reaction times (Hartley et al., 2019, Desrosiers et al., 2015)) can be observed at THC blood levels as low as 2–5 µg/L (Ramaekers et al., 2006), with risk of RTC following cannabis use estimated at an odds ratio of 1.28 (95% confidence interval 1.16–1.40) (Rogeberg, 2019). The median THC blood level in RTC fatalities in this study was 9 µg/L, which indicates probable cognitive impairment at the time of the incidents, when accounting for THC metabolism rates (Desrosiers et al., 2014; Hunault et al., 2010) and post-mortem redistribution (PMR) (Brunet et al., 2010; Holland et al., 2011; Yarema and Becker, 2005). Cannabis is thought to be highly susceptible to PMR due to its lipophilic nature and high volume of distribution (4–14 L/kg) (Yarema and Becker, 2005). In addition to the evident risk of fatal injury that this poses to the driver, potential for harm extends to passengers and others in the local vicinity (Chihuri and Li, 2020; Li et al., 2012; Martin et al., 2017). Guidance regarding the timeframe at which it can be deemed ‘safe’ to drive following cannabis use is, however, difficult to define due to variations in dose, dosage form, route of administration, interindividual metabolism and excretion (McCartney et al., 2021). Recent studies suggest that cannabis may affect driving performance up to 4–5 h following use (Marcotte et al., 2022, Arkell et al., 2020).

Cardiac failure was the most cited immediate cause of death following cannabis use in cannabis-only deaths, and the most commonly cited underlying physiological cause of death in polypharmacy cases. Cannabis has been found to have an impact on cardiovascular functioning, mainly in raising heart rate and blood pressure (Chetty et al., 2021, Jouanjus et al., 2017). Within the first hour after cannabis consumption there is an elevated risk of cannabis-associated myocardial infarction and an overall greater risk of mortality from myocardial infarction that increases with frequency of use (Desbois and Cacoub, 2013). Fatal cardiac events have been previously associated with cannabis use (Desai et al., 2018; Jouanjus et al., 2011), including in a recent study, which adjusted for variables that are independent predictors of heart failure (e.g. age, sex, diabetes mellitus, tobacco and alcohol use) (Kalla et al., 2018). Whilst the exact mechanism by which cannabis affects cardiac function is not fully understood, activation of CB1 receptors in cardiac smooth muscle can decrease contractility (Bonz et al., 2003), and there is evidence suggesting that regular cannabis use can induce structural and functional changes to cardiac chambers (Khanji et al., 2020).

Cannabis toxicity was cited as the sole (1a) cause of death (and therefore the immediate and underlying cause) in one case. The level of THC detected in post-mortem blood in this case (estimated 100–150 µg/L) far exceeds the median post-mortem THC blood concentration detected in this and a previously published study (Lemos and Ingle, 2011), and the median peak THC blood concentration detected in living users (Desrosiers et al., 2014). THC has been reported to persist at levels >5 µg/L in frequent users for over 30 h (Desrosiers et al., 2014), and this decedent was described as a very heavy cannabis user smoking multiple ‘joints’ a day. Such an elevated baseline THC blood level when coupled with extensive THC PMR may account for the high post-mortem blood level of THC detected in this case. However, it remains unclear as to the mechanism by which such a high THC concentration could cause fatal toxicity.

### Cannabis and fatal polydrug use

Whilst few cannabis users (<10%) report using other drugs simultaneously, cannabis is the most commonly co-administered drug in polydrug use scenarios (Home Office, 2015) – a trend reflected in this study as most deaths had at least one other psychoactive drug co-detected. The rise in polydrug use demonstrated in this study is a recognised growing problem both in the United Kingdom (Home Office, 2015), and abroad and may reflect the increased availability of drugs or an attempt of users to manage the undesirable effects of other drugs taken (Akhgari et al., 2021; Boileau-Falardeau et al., 2022; Connor et al., 2013; Golladay et al., 2020; Kandel et al., 2017; Lynskey et al., 2006). Fatal drug toxicity is a clear risk of polydrug use (Gudin et al., 2013), and is associated with other risky behaviours, such as intravenous drug use, which have clear links with increased mortality rate (Lorvick et al., 2018).

Opioids were co-detected in the largest proportion of cases in this study. Single substance non-fatal overdoses most frequently include opioids (both heroin and non-heroin opioids) (Liu and Vivolo-Kantor, 2020), and among polydrug non-fatal overdoses, alcohol, opioids, cannabis and cocaine feature in a large proportion of cases (Connor et al., 2013; Liu and Vivolo-Kantor, 2020; Lynskey et al., 2006; Smith et al., 2011). In this study cocaine co-detections had the largest increase of any co-detected substance, with 34% more cases reported in 2020 than in 2013. The purity of both powder and crack cocaine has concurrently risen in the United Kingdom (PHE, 2021), and a positive correlation between cocaine purity and emergency department visits has been observed (Zhu et al., 2014), which may explain in part the rise in fatalities with cocaine co-detections.

Although the prevalence of alcohol and cannabis co-detections remained relatively constant over time, the concomitant use of both alcohol and cannabis is harmful as these drugs act synergistically to heighten intoxication and behavioural impairment (Yurasek et al., 2017). Co-use of alcohol and cannabis is also associated with riskier driving behaviours than either drug alone (Ronen et al., 2010), and it increases the risk of fatal RTC (Chihuri et al., 2017).

### Cannabis potency has risen

The potency of both herbal cannabis and resin in the United Kingdom increased between 2009 and 2019 (EMCDDA, 2021; Potter et al., 2008, 2018), and it correlates with the median increase over time in detected THC levels in this study. Strong evidence for a relationship between amount and frequency of THC use with onset and severity of psychosis has been reported (Di Forti et al., 2019; Moore et al., 2007; Murray et al., 2016). The most seized cannabis form in the United Kingdom, sinsemilla (a dried plant material with typically higher potency than herbal cannabis), has a median THC content of 14.2% and virtually no cannabidiol (CBD) (<1%) (Potter et al., 2018). As CBD is reported to reduce psychotic effects induced by THC (Englund et al., 2013), sinsemilla lacks protection from this THC adverse effect. Cannabis users should reduce their intake to counteract the rising potency, avoid using with tobacco and other drugs, and potentially use preparations with higher CBD:THC ratios to reduce the risk of harm (Bourget, 2013; Englund et al., 2017; Kimbrel et al., 2018; Trott, 1992).

### Post-mortem cannabinoid quantifications have limited use in coronial investigations

THC concentrations following cannabis use are significantly higher in living users compared to those detected at post-mortem (Desrosiers et al., 2014; Holland et al., 2011; Huestis et al., 1992; Toennes et al., 2008). In addition, interindividual variability in administration technique (e.g. inhalation volume and frequency) and metabolism (Bergamaschi et al., 2013; Holland et al., 2011; Huestis et al., 1992; Karschner et al., 2009; Toennes et al., 2008) adds further complexity to the interpretation of cannabinoid levels and their relevance to cognitive impairment, impact on cardiac physiology or induction of psychosis. The presence or absence of cannabinoids in post-mortem toxicology testing may suffice in determining the cause and manner of death, and only have relevance in determining criminality with regards to drug-driving limits.

### Limitations

As NPSAD is reported to voluntarily, and post-mortem investigations with toxicology tests are not carried out for all deaths, the figures presented here likely under-represent the true number of deaths which have occurred in England where cannabinoids were present at post-mortem.

## Conclusion

The risk of death due to direct cannabis toxicity is negligible. However, there are clear harms associated with cannabis use that can prove fatal, including traumatic physical injury to self and others, and risk of cardiac complications. These indirect harms need careful consideration and further study to better elucidate the role cannabis plays in drug related mortality. Furthermore, the relevance of cannabinoid quantifications in determining cause of death in coronial investigations is limited.
